# Socioeconomic Inequities in Diet Quality and Nutrient Intakes among Australian Adults: Findings from a Nationally Representative Cross-Sectional Study

**DOI:** 10.3390/nu9101092

**Published:** 2017-10-04

**Authors:** Katherine M. Livingstone, Dana Lee Olstad, Rebecca M. Leech, Kylie Ball, Beth Meertens, Jane Potter, Xenia Cleanthous, Rachael Reynolds, Sarah A. McNaughton

**Affiliations:** 1 Geelong, Institute for Physical Activity and Nutrition (IPAN), School of Exercise and Nutrition Sciences, Deakin University, VIC 3220, Australia; rleec@deakin.edu.au (R.M.L.); kylie.ball@deakin.edu.au (K.B.); sarah.mcnaughton@deakin.edu.au (S.A.M.); 2 Department of Community Health Sciences, Cumming School of Medicine, University of Calgary, Calgary, AB T2N 4Z6, Canada; dana.olstad@ucalgary.ca (D.L.O.); Beth.meertens@heartfoundation.org.au (B.M.); 3 The National Heart Foundation of Australia, Melbourne, VIC 3000, Australia; Jane.Potter@heartfoundation.org.au (J.P.); Xenia.Cleanthous@csiro.au (X.C.); rachael.reynolds@news.com.au (R.R.)

**Keywords:** socioeconomic position, inequity, education, income, diet quality, nutrient intake

## Abstract

Poor diet may represent one pathway through which lower socioeconomic position (SEP) leads to adverse health outcomes. This study examined the associations between SEP and diet quality, its components, energy, and nutrients in a nationally representative sample of Australians. Dietary data from two 24-h recalls collected during the cross-sectional Australian Health Survey 2011-13 (*n* = 4875; aged ≥ 19 years) were analysed. Diet quality was evaluated using the Dietary Guidelines Index (DGI). SEP was assessed by index of area-level socioeconomic disadvantage, education level, and household income. Linear regression analyses investigated the associations between measures of SEP and dietary intakes. Across all of the SEP indicators, compared with the least disadvantaged group, the most disadvantaged group had 2.5–4.5 units lower DGI. A greater area-level disadvantage was associated with higher carbohydrate and total sugars intake. Lower education was associated with higher trans fat, carbohydrate, and total sugars intake and lower poly-unsaturated fat and fibre intake. Lower income was associated with lower total energy and protein intake and higher carbohydrate and trans fat intake. Lower SEP was generally associated with poorer diet quality and nutrient intakes, highlighting dietary inequities among Australian adults, and a need to develop policy that addresses these inequities.

## 1. Introduction

Lower socioeconomic position (SEP) has been associated with an increased risk of adverse health outcomes [1]. Poor diet is a strong modifiable risk factor for chronic disease [2]. Individuals with lower SEP, such as those with a lower level of education or income, or living in a socioeconomically disadvantaged neighbourhood, have poorer diets as compared with those with a higher SEP [3]. Specifically, dietary intakes amongst socioeconomically disadvantaged groups are typically characterised by a greater consumption of energy-dense foods [4], and lower intakes of fruits and vegetables [5], thereby resulting in poorer profiles of nutrient intakes [3].

Given that foods and nutrients are not consumed in isolation, the development of dietary recommendations requires consideration of the whole diet, or diet quality [6]. However, understanding of how SEP relates to overall diet quality and the intake of specific foods and nutrients is limited. Most studies have investigated associations between SEP and single foods and nutrients [7,8,9,10,11], while few studies have examined overall diet quality [3,12]. A recent repeat cross-sectional analysis in a nationally representative sample of 33,932 US adults identified worsening disparities in diet quality and some food and nutrient intakes by ethnicity, education, and income from 1999 to 2012 [3].

Some previous estimates of dietary intakes have been based on food frequency questionnaires [12] or a single dietary recall [10], which may be less representative of usual intakes when compared with two dietary recalls. Additionally, non-nationally-representative research has under-represented individuals from lower SEP groups, with little examination of how associations between SEP and diet differ by sex [12]. This is important since, in the same way that associations between SEP and obesity differ among men and women [13], the impact of SEP on diet may be sex-specific. The aims of this analysis were to investigate associations between SEP (area-level disadvantage, education level, and income) and dietary intakes (diet quality and food group, energy and nutrient intakes) and to evaluate how these relationships differ by sex, in a nationally representative sample of Australian adults.

## 2. Materials and Methods 

### 2.1. Study Design and Participants

The present analyses were based on data provided by adults (≥19 years; *n* = 4875) collected during the National Nutrition and Physical Activity Survey (NNPAS) component of the 2011-13 Australian Health Survey. The NNPAS is a population-based survey administered by the Australian Bureau of Statistics (ABS) that sampled households in urban and rural areas across all Australian states and territories [14]. A total of 14,363 private dwellings were selected, of which 9519 households (77.0% response rate; *n* = 12,153 individuals) responded to the first interview. Dietary intakes were estimated using two, 24-h dietary recalls. For the present analysis, participants were excluded if they (i) were pregnant and/or breastfeeding; (ii) had missing data for outcomes or covariates; and, (iii) had only one day of dietary recall (Figure S1). NNPAS questionnaires were administered under the authority of the Census and Statistics Act 1905.

### 2.2. Area-Level Disadvantage 

Area-level disadvantage was assessed using the ABS Socio-Economic Indexes for Areas (SEIFA). SEIFA includes four indices of disadvantage, of which the 2006 Index of Relative Socio-Economic Disadvantage was the measure of SEIFA used in this analysis. This index of area-level disadvantage ranked Australian areas according to relative socioeconomic disadvantage, combining attributes such as low income, low educational attainment, high unemployment, and jobs in relatively unskilled occupations [14]. Area-level disadvantage was divided into quintiles ranging from the least disadvantaged (i.e., most affluent—quintile 5) to the most disadvantaged (quintile 1).

### 2.3. Education Level

Education level was derived from two questions based on the Australian Standard Classification of Education 2001 [14]. This was operationalized as low (completed some high school or less), medium (completed high school or completed some high school and/or certificate/diploma), and high (University qualification).

### 2.4. Household Income

Income was ascertained by asking participants the combined income (from all sources) of all household members aged 18 years and over. Deciles of participant weekly gross household income, taking into account the number of persons living in the household (termed ‘equivalised income’), were estimated [15]. Deciles were collapsed into quintiles and expressed in Australian dollars per week: highest 20% (≥$1152); Q2 ($959–1151); Q3 ($639–958); Q4 ($399–638); and, lowest 20% (below poverty line; ≤$398).

### 2.5. Dietary Intake

An automated, multiple-pass, 24-h dietary recall was used to provide quantitative information on foods and beverages consumed on the day prior to interview based on the USDA Automated Multiple-Pass Method [16]. A second 24-h recall, via telephone interview, was collected at least eight days after the first interview. Given that the aim of this paper was to examine associations between variables, rather than to determine the prevalence of adequate/inadequate intakes, and our results are aligned with previous findings using the AHS data [17], data from those participants who completed both recalls (65%) were included in the present analysis and an average of both days was used. Nutrient intakes (total energy (KJ/day), percentage energy from total fat, saturated fat (SFA), mono-unsaturated fat (MUFA), poly-unsaturated fat (PUFA), trans fat, carbohydrates, total sugars and protein, and fibre and sodium density (g/MJ) were derived from the 24-h recalls using the Australian Supplement and Nutrient Database 2011-13 [18]. Information on usual daily use of salt at the table and during cooking (ranging from “Very often” to “Not used”) was collected using brief questionnaire items [14].

### 2.6. Dietary Guideline Index

The Dietary Guidelines Index (DGI) is a food-based score designed to reflect the diet quality of individuals according to compliance with the 2013 Australian Dietary Guidelines [19]. Dietary intakes of individuals, based on 24-h recalls and brief questionnaire items, were scored according to ten recommended dietary components (food variety, fruit, vegetables, cereals, meat and alternatives, dairy and alternatives, and fluid intake) and six discouraged dietary components (discretionary foods, SFA, unsaturated fat, added salt, extra sugar, and alcohol). Further details on foods included in each component are available elsewhere [17].

DGI scores ranged between 0 and 130, with a higher score indicating a better diet quality (Table S1). Each item was scored out of 10, with 0 indicating that the guideline was not met. Cut-offs used to obtain the maximum score for each component were tailored to age- and sex-specific food-based recommendations outlined in the Australian Dietary Guidelines [20]. Proportionate scores were derived where intakes fell between the maximum and minimum scoring criteria for all of the items except discretionary foods, saturated and unsaturated fat, salt, sugar, and alcohol, which scored either 0 or 10 [17,21,22].

### 2.7. Covariates

Covariates were selected from existing literature [12]. Smoking habits (current smoker, ex-smoker, or never smoked) and remoteness classification (major city in Australia, inner regional Australia, or other) were collected via an interviewer-administered questionnaire [14]. Energy misreporting was calculated as the ratio of reported total energy intake to predicted total energy expenditure (EI:EE) using sex- and age-specific equations for a range of weight statuses, assuming a “low active” physical activity level (≥1.4 < 1.6). [23,24] Participants were identified as plausible, under or over reporters of energy intake using published equations to calculate the ±1 standard deviation (SD) cut-off for EI:EE [23].

### 2.8. Statistical Analyses

Multiple linear regression was used to test for significant differences between measures of SEP (area-level disadvantage, education, and income (categorical); independent variable) and dietary intake (DGI, DGI component scores, and total energy and nutrient intakes (continuous); dependent variables). Analyses were adjusted for age (continuous), sex (except when used to stratify), smoking (categorical), remoteness classification (categorical), and EI:EE (continuous). Likelihood ratio chi-square tests were used to confirm that SEP could be treated as a continuous variable in regression models examining the relationship between SEP and DGI (data not shown). All analyses were also stratified by sex. Sensitivity analyses were performed to examine the associations between SEP and EI:EE and between SEP and dietary intakes following the exclusion of energy intake misreporters. As a sensitivity analysis, raw intakes were used rather than the component scores to avoid truncation once converted to scores between 0 and 10.

Data were analysed using Stata (version 14; StataCorp., College Station, TX, USA) applying survey weightings to account for the survey design and for the probability of selection. *p* < 0.05 was considered statistically significant. Given that our hypothesis was predefined (i.e., that lower SEP would be associated with poorer diet, at the level of nutrients, diet quality and its components) no adjustments were made for multiple comparisons [25].

## 3. Results

A total of 4875 individuals (47% women) were included in the present analyses. Characteristics of omitted individuals were broadly similar, although slightly more females, younger adults, not married individuals, and individuals living in major cities were omitted based on missing covariates than based on missing a second dietary recall. Of the individuals included, 47% were female, 63% were married, and 70% and 44% were living in major cities and in households with families, respectively. Twenty four percent had a high level of education. Participant characteristics and dietary intakes by area-level disadvantage, education level, and income are presented in Tables S2 and S3.

### 3.1. Diet Quality and DGI Component Scores by Socioeconomic Position

**Figure 1:**
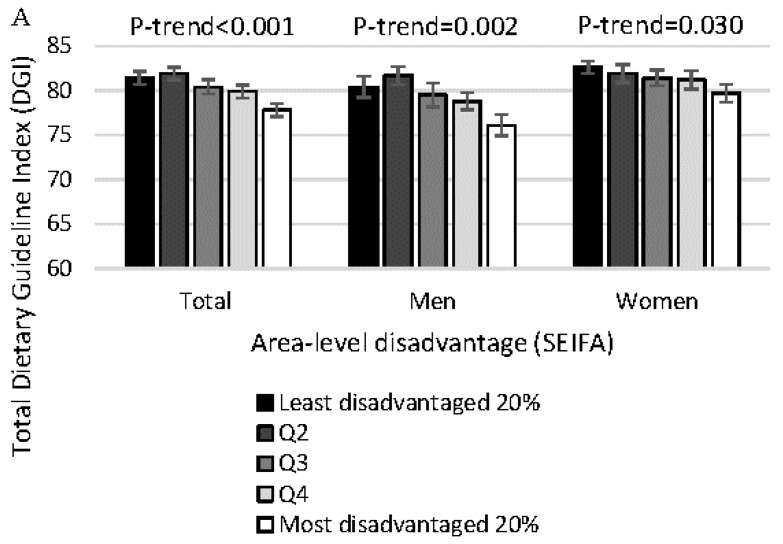

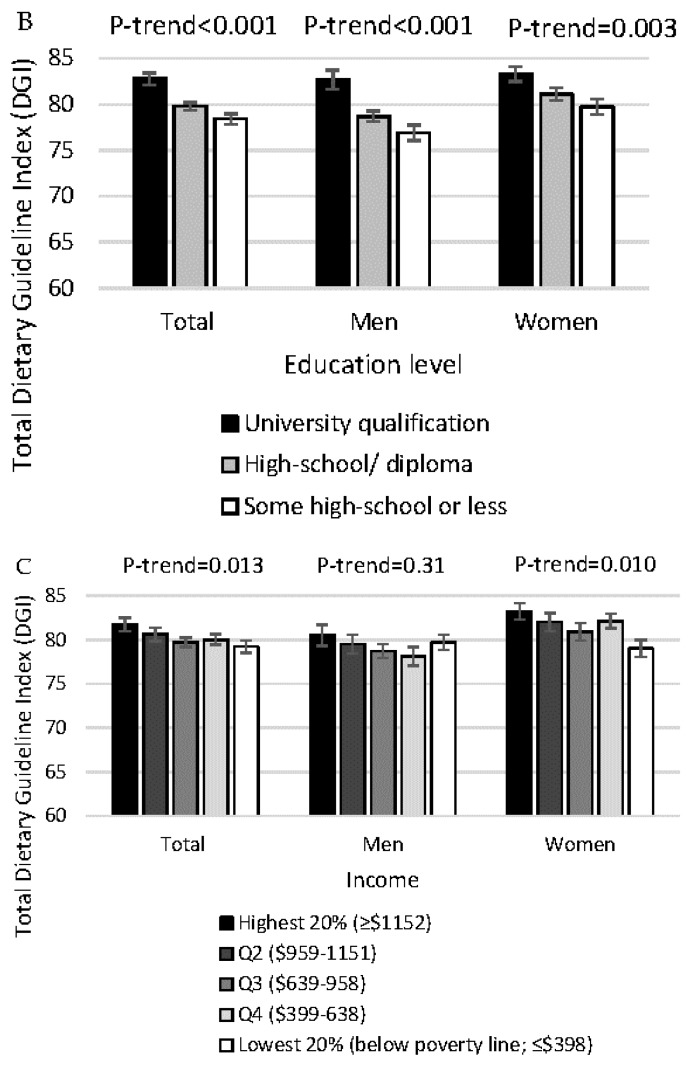
Dietary Guideline Index (DGI) in men (*n* = 2356) and women (*n* = 2519) from the 2011-13 Australian National Nutrition and Physical Activity Survey by: (**A**) area-level disadvantage; (**B**) education; and, (**C**) income. Area-level disadvantage was assessed using SEIFA (Socio-Economic Index for Areas); Q, quintile; DGI scores could range between 0 and 130. Values represent predictive margins and SE.

As shown in Figure 1, lower SEP was associated with 2.5–4.5 units lower DGI, depending on the SEP indicator. With greater area-level disadvantage, individuals had up to 3.6 units lower DGI scores. As shown in Table 1 and Table 2, greater area-level disadvantage was associated with lower DGI component scores (i.e., poorer adherence to dietary recommendations) for 6 of the 13 components (Table 1). Individuals with lower education had up to 4.5 units lower DGI as compared with those with a higher education. Lower education was also associated with lower DGI component scores for 9 of the 13 components (Table 2). Across higher quintiles of income, individuals had up to 2.5 units lower DGI scores. Lower income was associated with lower DGI component scores for 6 of the 13 components (Table 2).

### 3.2. Total Energy and Nutrient Intakes by Socioeconomic Position

A greater area-level disadvantage was associated with lower total fat and higher carbohydrate and total sugars intake, while there was a trend towards lower SFA intake (Table 3 and Table 4). Area-level disadvantage was not significantly associated with total energy, protein, MUFA, PUFA, fibre, or sodium intake. Lower education was associated with higher trans fat, carbohydrate, and total sugars intake, and lower PUFA and fibre intake. Education level was not significantly associated with total energy, protein, total fat, SFA, MUFA, or sodium intake. Lower quintiles of income were associated with lower total energy, and protein intake and higher trans fat and carbohydrate intake. Income was not significantly associated with total fat, SFA, MUFA, PUFA, total sugars, fibre, or sodium intake.

### 3.3. Stratified Analyses by Sex

As shown in stratified analyses (Table 1, Table 2, Table 3 and Table 4), associations between SEP and DGI, DGI component scores and nutrient intakes differed between males and females. Greater area-level disadvantage was associated with lower cereal and added salt scores in men and lower fluid scores in women. Greater area-level disadvantage was associated with a lower total fat and MUFA intake and higher total sugars intake in women. Lower education was associated with lower vegetable, cereal, and discretionary food scores, and higher total sugars intake in men; and with lower PUFA and higher carbohydrate intake in women. Lower income was associated with lower DGI scores, lower scores for vegetables, cereals, proportion of lean meat and fluid scores, higher trans fat, and lower MUFA intake in women. Lower income was associated with lower scores for proportion of water intake and higher scores for alcohol intake in men.

### 3.4. Sensitivity Analyses

Greater area-level disadvantage (coeff −0.01, SE 0.01; *p*-trend = 0.038) and lower education (coeff −0.04, SE 0.01; *p*-trend < 0.001) and income (coeff −0.01, SE 0.01; *p*-trend = 0.018) were associated with higher energy misreporting. Characteristics of plausible energy reporters and the total sample are compared in Table S4. Energy misreporters (*n* = 1555) were excluded from sensitivity analyses, with the following results reporting any differences following these exclusions. Area-level disadvantage was no longer associated with total cereal and total fluid component scores. Greater area-level disadvantage became associated with lower protein (coef: −0.23, SE 0.10; *p*-trend = 0.019) and MUFA intake (coef: −0.14, SE 0.06; *p*-trend = 0.031). Education level was no longer associated with component scores for cereals, fluid, and added salt at the table. Lower education became associated with lower total energy (−166.3, 65.8; *p*-trend = 0.014) and total fat intake (−0.39, 0.14; *p*-trend = 0.006), but was no longer associated with fibre intake. Income was no longer associated with DGI or with fruit, vegetables, discretionary foods, and total added salt component scores. Income was no longer associated with trans fat intake. Associations between SEP and DGI component scores were consistent when examined as raw intakes.

## 4. Discussion

The present study is one of the first nationally-representative studies in Australia to evaluate the associations between SEP and dietary intakes at the level of nutrients, foods, and overall diet quality based on two days of dietary recalls. Despite decades of evidence of socioeconomic inequities in dietary intakes worldwide, our findings confirm that poorer diets in lower socioeconomic groups persist. Importantly, these inequities are present not just for individual nutrients and foods, but for overall diet quality as well.

Our findings are comparable with other studies that have investigated the associations between SEP and diet quality [3,12]. In a non-representative sample of 9294 adults aged 25 years and older from the 1999–2000 Australian Diabetes and Lifestyle Study, higher education, income, and lower area-level disadvantage were associated with 2–5 units higher DGI [12]. These unit differences are in line with the present study, which examined DGI scores that reflect compliance with the most recent (2013) dietary guidelines. For DGI component scores, we observed higher scores for fruits, vegetables, cereals (serves/day), and dairy and alternatives with higher education, and higher scores for meat and alternatives with higher income, which is consistent with recent findings from the US National Health and Nutrition Examination Survey, where less marked increases in intakes of these foods were observed for individuals with lower education and income [3]. However, lack of associations observed between area-level disadvantage and income and fruit and vegetables, as well as between all of the measures of SEP and DGI components that should be limited (e.g., foods high in saturated fat), warrants further investigation, given the consistent associations of SEP with fruit and vegetable intake in previous analyses [26]. Examination of how diet quality and food intakes change over time according to SEP is also warranted [3].

The strength and gradation of the relationships between SEP and diet quality varied by sex. For example, income was associated with diet quality in women only. Previous research indicates that diet quality is lower in men than women [27], which is consistent with research showing that women are more health-conscious and that their diets better align with dietary guidelines [28]. This difference in diet quality score by sex may have contributed to the lack of association with income. Given that the three indicators of SEP assess different underlying constructs, and that we observed differential findings in the present study, future studies should give careful consideration to which construct is most appropriate to the study aims, or should include multiple measures [29].

Our findings for an association between lower total energy intake and lower income, but not area-level disadvantage or education, is consistent with previous research [30,31]. These findings, although limited to one SEP indicator, may be explained by higher energy misreporting in lower SEP groups, as was found in the present study [32]. Although participant characteristics and relationships between SEP and diet were similar following the exclusion of misreporters, income was no longer associated with DGI, reinforcing consideration of the differing applicability of SEP indicators. Moreover, loss of significant associations following exclusion of energy misreporters warrants further investigation given that a comparison of participant characteristics in the total sample and the valid reporters showed minimal sample differences. Nonetheless, despite extensive consideration of energy misreporting in our analyses, residual confounding cannot be discounted.

The evidence in relation to associations between SEP and nutrient intakes is mixed [33]. Consistent with previous research, we observed that lower education was associated with lower fibre intake [34]. However, this was not consistent for area-level disadvantage or income. In addition, we observed no significant associations between SEP and sodium intake. Some dietary components, such as sodium, have been poorly correlated with their urinary metabolites [35]. As a result, the present dietary recall may be limited in its ability to accurately measure some nutrient intakes. Nevertheless, the mixed findings for the associations between SEP and nutrient intakes, as well as DGI components, may demonstrate the conflicting mechanisms through which SEP influences diet. As a result, this study highlights the benefits of evaluating diet as a whole for improving the interpretability of the findings.

This study has a number of strengths. Given that this study was conducted in a large, nationally representative sample, our results should be representative of the breadth of SEP in the Australian population. Our diet quality score was derived from two dietary recalls using age- and sex-specific cut-offs for component scores, thus offering a more comprehensive and reliable estimate of dietary intake than previous estimates of diet quality based on food frequency questionnaires or a single dietary recall. A further strength of this study is that we compared three established indicators of SEP, thereby providing a more complete picture of dietary inequities.

Despite the strengths of this study, certain limitations should be acknowledged. Due to the cross-sectional design we were unable to infer any temporal relationships between SEP and dietary intakes, and thus prospective studies are warranted. Missing data, most notably for income and the second day of dietary recalls, may have introduced bias. However, survey weighting that accounted for biases associated with the second day of dietary recall may help mitigate biases associated with dietary recalls. Numerous statistical tests were undertaken without adjustment for multiple comparisons, thus some statistically significant findings may be false-positive results due to type I error.

The present study has implications for the design of future nutrition promotion strategies, as well as highlighting the importance of social determinants of the nutrition and health status of all Australians. We provide consistent evidence for SEP-based gradients in diet quality. Specifically, 2.5–4.5 unit higher DGI with higher SEP seen in the present study is equivalent to an extra serve of fruit or lean meat daily, or less frequent use of salt. These differences in serves, if sustained, could have significant effects on health outcomes, such as obesity and cardiovascular disease [36], and thus have important public health implications. Given that macronutrient and DGI-subcomponent displacement was not evaluated in this study, the implications of any differences in these is less clear and should be interpreted with caution. For example, whether any downward trend in %energy from saturated fat was associated with a corresponding upward trend in %energy from sugar. Moreover, some of these differences may be small and hard to interpret. Our findings support the need to develop interventions that address dietary behaviours across multiple levels of SEP, but with a specific focus on lower SEP groups. Such interventions can improve on “one size fits all” dietary recommendations, which may be primarily effective in higher SEP groups [37]. Additionally, interventions that seek to address the underlying social determinants of health inequities are likely to result in improvements in dietary behaviors across the socio-economic gradient.

## 5. Conclusions

Lower SEP was associated with lower diet quality and poorer intakes of some foods and nutrients in a nationally representative sample of Australian adults. In addition, these relationships differed by sex. This research adds contemporary data to the growing body of evidence on dietary inequities in Australia. Given that these dietary inequities may increase the risk of obesity and chronic disease in disadvantaged groups, these findings have important implications for the development of policy to address these inequities. Specifically, gender-tailored healthy eating strategies that are sensitive to the needs of people experiencing socioeconomic disadvantage are needed.

## Figures and Tables

**Table 1:** Dietary Guideline Index (DGI) sub-components by area-level disadvantage in men (*n* = 2356) and women (*n* = 2519) from the 2011-13 Australian National Nutrition and Physical Activity Survey.

DGI sub-components	Sample	Area-Level Disadvantage					
		Least Disadvantaged 20% ^1^	Q2	Q3	Q4	Most Disadvantaged 20%	*p*-*Trend* ^2^
1. Food variety	Overall	3.68 (0.08)	3.62 (0.07)	3.46 (0.07)	3.41 (0.07)	3.18 (0.07)	**<0.001**
	Men	3.47 (0.14)	3.53 (0.10)	3.39 (0.10)	3.24 (0.12)	3.01 (0.12)	**0.007**
	Women	3.91 (0.09)	3.71 (0.11)	3.55 (0.10)	3.60 (0.09)	3.37 (0.08)	**<0.001**
2. Vegetables	Overall	4.88 (0.13)	4.79 (0.12)	4.93 (0.15)	4.85 (0.14)	4.77 (0.15)	0.70
	Men	4.61 (0.18)	4.52 (0.16)	4.69 (0.26)	4.66 (0.17)	4.89 (0.20)	0.27
	Women	5.16 (0.19)	5.14 (0.20)	5.17 (0.20)	5.11 (0.21)	4.67 (0.21)	0.14
3. Fruit	Overall	5.49 (0.20)	6.05 (0.15)	5.62 (0.21)	5.65 (0.18)	5.18 (0.18)	0.18
	Men	5.37 (0.27)	5.94 (0.21)	5.45 (0.31)	5.64 (0.26)	4.92 (0.33)	0.29
	Women	5.63 (0.22)	6.18 (0.24)	5.84 (0.25)	5.62 (0.20)	5.46 (0.21)	0.30
4. Cereal (total)	Overall	5.37 (0.15)	5.67 (0.13)	5.20 (0.16)	5.47 (0.12)	5.11 (0.12)	0.14
	Men	5.54 (0.19)	5.87 (0.16)	5.44 (0.21)	5.49 (0.19)	4.85 (0.19)	**0.012**
	Women	5.17 (0.19)	5.43 (0.20)	4.92 (0.21)	5.41 (0.16)	5.40 (0.20)	0.41
4a. Serves per day	Overall	2.87 (0.06)	2.88 (0.06)	2.91 (0.05)	2.99 (0.07)	3.08 (0.08)	**0.022**
	Men	3.24 (0.90)	3.13 (0.07)	3.10 (0.8)	3.12 (0.10)	3.11 (0.09)	0.37
	Women	2.47 (0.07)	2.58 (0.08)	2.71 (0.09)	2.82 (0.09)	3.04 (0.12)	**<0.001**
4b. Mostly wholegrain	Overall	2.35 (0.14)	2.60 (0.10)	2.12 (0.13)	2.31 (0.11)	1.93 (0.10)	**0.010**
	Men	1.23 (0.17)	2.59 (0.14)	2.22 (0.18)	2.19 (0.17)	1.67 (0.17)	**0.013**
	Women	1.48 (0.18)	2.62 (0.18)	2.00 (0.20)	2.42 (0.14)	2.25 (0.16)	0.27
5. Meat and alternatives (total)	Overall	8.23 (0.10)	8.09 (0.11)	7.89 (0.11)	7.81 (0.09)	7.66 (0.13)	**0.001**
	Men	8.32 (0.12)	8.04 (0.17)	7.79 (0.17)	7.78 (0.16)	7.73 (0.20)	**0.010**
	Women	8.13 (0.14)	8.16 (0.14)	7.99 (0.14)	7.84 (0.11)	7.61 (0.25)	**0.042**
5a. Serves per day^3^	Overall	3.47 (0.07)	3.37 (0.08)	3.23 (0.08)	3.18 (0.07)	3.11 (0.07)	**0.001**
	Men	3.59 (0.09)	3.40 (0.12)	3.22 (0.13)	3.20 (0.12)	3.15 (0.14)	**0.004**
	Women	3.34 (0.10)	3.34 (0.11)	3.23 (0.09)	3.15 (0.08)	3.06 (0.13)	**0.042**
5b. Mostly lean^3^	Overall	4.76 (0.04)	4.72 (0.05)	4.66 (0.05)	4.64 (0.04)	4.56 (0.07)	**0.015**
	Men	4.73 (0.05)	4.63 (0.07)	4.57 (0.07)	4.58 (0.07)	4.58 (0.09)	0.13
	Women	4.80 (0.06)	4.82 (0.04)	4.75 (0.07)	4.69 (0.05)	4.55 (0.14)	0.09
6. Total dairy and alternatives	Overall	5.27 (0.16)	5.20 (0.14)	4.90 (0.16)	4.70 (0.16)	4.53 (0.15)	**<0.001**
	Men	5.65 (0.22)	5.88 (0.20)	5.36 (0.21)	5.05 (0.26)	4.84 (0.25)	**0.002**
	Women	4.84 (0.20)	4.41 (0.17)	4.38 (0.22)	4.28 (0.17)	4.16 (0.18)	**0.010**
7. Fluid intake (total)	Overall	8.45 (0.09)	8.62 (0.10)	8.44 (0.11)	8.49 (0.08)	8.18 (0.12)	0.06
	Men	8.04 (0.14)	8.47 (0.12)	8.04 (0.21)	8.17 (0.12)	7.95 (0.15)	0.38
	Women	8.93 (0.08)	8.77 (0.11)	8.90 (0.10)	8.83 (0.11)	8.45 (0.16)	**0.034**
7a. Serves per day	Overall	3.96 (0.06)	4.07 (0.07)	3.90 (0.08)	3.87 (0.06)	3.73 (0.08)	**0.012**
	Men	3.66 (0.10)	3.95 (0.09)	3.62 (0.13)	3.58 (0.09)	3.51 (0.11)	0.07
	Women	4.30 (0.06)	4.18 (0.07)	4.24 (0.08)	4.19 (0.08)	4.00 (0.10)	**0.030**
7b. Mostly water	Overall	4.50 (0.05)	4.56 (0.05)	4.53 (0.06)	4.62 (0.04)	4.45 (0.07)	0.93
	Men	4.38 (0.07)	4.52 (0.06)	4.42 (0.11)	4.58 (0.06)	4.45 (0.10)	0.40
	Women	4.62 (0.05)	4.59 (0.06)	4.66 (0.05)	4.65 (0.06)	4.45 (0.09)	0.21
8. Limit discretionary foods	Overall	2.38 (0.21)	2.93 (0.23)	2.76 (0.21)	2.72 (0.20)	2.64 (0.23)	0.59
	Men	2.16 (0.29)	2.61 (0.20)	2.38 (0.31)	2.40 (0.27)	2.13 (0.32)	0.86
	Women	2.66 (0.27)	3.25 (0.29)	3.24 (0.31)	3.07 (0.29)	3.20 (0.32)	0.29
9. Limit saturated fat (total)	Overall	8.63 (0.11)	8.58 (0.10)	8.81 (0.10)	8.70 (0.10)	8.72 (0.12)	0.43
	Men	8.38 (0.19)	8.56 (0.14)	8.76 (0.15)	8.54 (0.13)	8.76 (0.14)	0.18
	Women	8.88 (0.10)	8.57 (0.17)	8.86 (0.14)	8.91 (0.15)	8.68 (0.18)	0.84
9a. Mostly trimmed meat	Overall	4.04 (0.07)	4.00 (0.07)	4.13 (0.07)	4.09 (0.07)	4.17 (0.08)	0.13
	Men	3.96 (0.12)	3.98 (0.11)	4.06 (0.11)	3.87 (0.09)	4.13 (0.11)	0.52
	Women	4.14 (0.08)	4.00 (0.13)	4.21 (0.09)	4.35 (0.09)	4.21 (0.09)	0.09
9b. Mostly low-fat milk	Overall	4.60 (0.06)	4.59 (0.06)	4.66 (0.06)	4.55 (0.07)	4.55 (0.08)	0.54
	Men	4.42 (0.10)	4.58 (0.09)	4.69 (0.08)	4.55 (0.09)	4.59 (0.10)	0.32
	Women	4.78 (0.06)	4.59 (0.07)	4.63 (0.10)	4.57 (0.09)	4.49 (0.12)	0.06
10. Moderate unsaturated-fat	Overall	8.36 (0.13)	7.84 (0.23)	8.33 (0.18)	8.45 (0.18)	8.49 (0.20)	0.17
	Men	9.07 (0.15)	8.56 (0.23)	9.04 (0.18)	9.00 (0.16)	8.88 (0.29)	0.99
	Women	7.59 (0.23)	6.97 (0.37)	7.58 (0.34)	7.79 (0.34)	8.02 (0.31)	0.09
11. Limit added salt (total)	Overall	6.17 (0.16)	6.18 (0.15)	5.90 (0.14)	5.78 (0.15)	5.54 (0.16)	**<0.001**
	Men	6.03 (0.22)	6.08 (0.21)	5.74 (0.21)	5.65 (0.21)	5.28 (0.22)	**0.001**
	Women	6.31 (0.24)	6.29 (0.18)	6.04 (0.20)	6.00 (0.18)	5.84 (0.20)	0.07
11a. During cooking	Overall	2.97 (0.11)	2.81 (0.11)	2.61 (0.98)	2.41 (0.12)	2.32 (0.13)	**<0.001**
	Men	2.88 (0.17)	2.82 (0.17)	2.62 (0.15)	2.34 (0.15)	2.28 (0.16)	**0.001**
	Women	3.05 (0.15)	2.79 (0.11)	2.57 (0.14)	2.56 (0.15)	2.37 (0.17)	**0.001**
11b. Added at the table	Overall	3.20 (0.11)	3.37 (0.10)	3.29 (0.09)	3.37 (0.08)	3.22 (0.10)	0.81
	Men	3.15 (0.15)	3.26 (0.13)	3.11 (0.13)	3.31 (0.12)	3.00 (0.14)	0.64
	Women	3.26 (0.14)	3.50 (0.13)	3.48 (0.14)	1.45 (0.12)	3.47 (0.13)	0.38
12. Limit extra sugar	Overall	6.91 (0.25)	6.44 (0.22)	6.47 (0.16)	6.39 (0.23)	6.42 (0.23)	0.14
	Men	6.66 (0.34)	6.11 (0.35)	6.02 (0.33)	6.32 (0.33)	6.17 (0.35)	0.51
	Women	7.18 (0.29)	6.83 (0.33)	6.94 (0.21)	6.48 (0.29)	6.73 (0.33)	0.18
13. Limit alcohol	Overall	8.45 (0.18)	8.79 (0.15)	8.58 (0.17)	8.66 (0.15)	8.34 (0.17)	0.62
	Men	7.89 (0.31)	8.43 (0.23)	8.23 (0.28)	8.07 (0.26)	7.68 (0.26)	0.47
	Women	9.08 (0.16)	9.21 (0.15)	9.00 (0.20)	9.29 (0.16)	9.10 (0.21)	0.79

Area-level disadvantage was assessed using SEIFA (Socio-Economic Index for Areas); Q, quintile; DGI scores could range between 0 and 130. Values represent predictive margins and SE. ^1^ Denotes group of lowest socioeconomic disadvantage; ^2^ Linear regression analyses were adjusted for age, sex (not when used to stratify), urban/rural location, smoking and ratio of energy intake to predicted energy expenditure (EI:EE).

**Table 2:** Dietary Guideline Index (DGI) sub-components by education and income in men (*n* = 2356) and women (*n* = 2519) from the 2011-13 Australian National Nutrition and Physical Activity Survey.

DGI sub-components	Sample	Education				Gross Equivalised Income of Household (Weekly)					
		University Qualification ^1^	High-School/Diploma	Some High-School or Less	*p*-*Trend^2^*	Highest 20% (≥$1152) ^1^	Q2 ($959–1151)	Q3 ($639–958)	Q4 ($399–638)	Lowest 20% (Below Poverty Line; ≤$398)	*p*-*Trend* ^2^
1. Food variety	Overall	3.73 (0.07)	3.43 (0.06)	3.26 (0.06)	<0.001	3.80 (0.07)	3.61 (0.08)	3.39 (0.08)	3.38 (0.08)	3.08 (0.07)	**<0.001**
	Men	3.65 (0.10)	3.27 (0.07)	3.12 (0.09)	0.001	3.57 (0.10)	3.45 (0.11)	3.32 (0.12)	3.13 (0.11)	3.02 (0.12)	**0.001**
	Women	3.84 (0.09)	3.63 (0.07)	3.42 (0.08)	0.001	4.07 (0.11)	3.79 (0.10)	3.47 (0.10)	3.67 (0.10)	3.12 (0.10)	**<0.001**
2. Vegetables	Overall	5.26 (0.13)	4.67 (0.09)	4.72 (0.11)	0.002	5.20 (0.13)	4.86 (0.13)	4.56 (0.12)	4.63 (0.15)	4.95 (0.16)	0.07
	Men	5.17 (0.21)	4.46 (0.12)	4.59 (0.17)	0.021	4.93 (0.21)	4.58 (0.19)	4.42 (0.17)	4.43 (0.23)	4.99 (0.22)	0.65
	Women	5.34 (0.18)	4.91 (0.15)	4.97 (0.15)	0.10	5.50 (0.21)	5.17 (0.17)	4.71 (0.20)	4.88 (0.19)	5.01 (0.21)	**0.035**
3. Fruit	Overall	6.07 (0.17)	5.55 (0.11)	5.17 (0.18)	0.002	5.68 (0.15)	5.76 (0.15)	5.39 (0.16)	5.94 (0.21)	5.22 (0.18)	0.15
	Men	5.92 (0.27)	5.45 (0.16)	4.97 (0.29)	0.029	5.46 (0.21)	5.66 (0.26)	5.17 (0.25)	5.90 (0.32)	5.18 (0.27)	0.72
	Women	6.23 (0.21)	5.63 (0.13)	5.39 (0.23)	0.018	5.96 (0.20)	5.88 (0.20)	5.65 (0.22)	6.00 (0.22)	5.29 (0.27)	0.10
4. Cereal (total)	Overall	5.58 (0.12)	5.29 (0.09)	5.23 (0.13)	0.10	5.38 (0.15)	5.45 (0.13)	5.45 (0.15)	5.35 (0.10)	5.13 (0.15)	0.30
	Men	5.85 (0.16)	5.33 (0.10)	5.26 (0.22)	0.023	5.52 (0.20)	5.48 (0.17)	5.59 (0.22)	5.27 (0.16)	5.27 (0.23)	0.41
	Women	5.32 (0.18)	5.28 (0.12)	5.17 (0.13)	0.56	51.9 (0.19)	5.42 (0.20)	5.28 (0.17)	5.43 (0.18)	4.98 (0.21)	0.51
4a. Serves per day	Overall	2.85 (0.05)	2.94 (0.04)	3.05 (0.07)	0.033	2.73 (0.05)	2.94 (0.06)	2.94 (0.06)	3.07 (0.07)	3.11 (0.07)	**<0.001**
	Men	3.17 (0.06)	3.12 (0.05)	3.17 (0.10)	0.94	3.03 (0.08)	3.13 (0.08)	3.15 (0.08)	3.20 (0.80)	3.27 (0.11)	0.11
	Women	2.55 (0.07)	2.77 (0.05)	2.81 (0.09)	0.028	2.38 (0.09)	2.71 (0.10)	2.70 (0.09)	2.91 90.09)	2.89 (0.08)	**<0.001**
4b. Mostly wholegrain	Overall	2.56 (0.10)	2.20 (0.07)	2.04 (0.11)	0.002	2.46 (0.14)	2.39 (0.12)	2.38 (0.13)	2.10 (0.09)	1.86 (0.12)	**0.001**
	Men	2.56 (0.14)	2.09 (0.09)	1.97 (0.18)	0.007	2.38 (0.18)	2.24 (0.15)	2.34 (0.18)	1.88 (0.15)	1.90 (0.20)	**0.048**
	Women	2.57 (0.15)	2.34 (0.11)	2.13 (0.11)	0.039	2.52 (0.18)	2.56 (0.17)	2.43 (0.15)	2.36 (0.15)	1.87 (0.18)	**0.016**
5. Meat and alternatives (total)	Overall	7.96 (0.10)	7.99 (0.07)	7.84 (0.10)	0.48	8.39 (0.09)	7.85 (0.13)	7.96 (0.10)	7.86 (0.13)	7.56 (0.10)	**<0.001**
	Men	7.94 (0.13)	7.95 (0.11)	7.92 (0.17)	0.96	8.38 (0.13)	7.78 (0.16)	7.90 (0.15)	7.81 (0.19)	7.62 (0.14)	**0.002**
	Women	7.98 (0.12)	8.06 (0.08)	7.76 (0.12)	0.20	8.40 (0.10)	7.92 (0.21)	8.01 (0.13)	7.93 (0.17)	7.52 (0.13)	**<0.001**
5a. Serves per day	Overall	3.26 (0.06)	3.32 (0.05)	3.21 (0.06)	0.71	3.58 (0.07)	3.26 (0.08)	3.27 (0.08)	3.22 (0.08)	2.98 (0.06)	**<0.001**
	Men	3.28 (0.09)	3.34 (0.07)	3.32 (0.11)	0.79	3.65 (0.09)	3.24 (0.10)	3.30 (0.09)	3.20 (0.14)	3.04 (0.09)	**<0.001**
	Women	3.23 (0.08)	3.31 (0.06)	3.11 (0.08)	0.36	3.48 (0.09)	3.28 (0.12)	3.22 (0.10)	3.24 (0.10)	2.93 (0.10)	**<0.001**
5b. Mostly lean	Overall	4.70 (0.05)	4.67 (0.03)	4.62 (0.05)	0.30	4.81 (0.03)	4.59 (0.07)	4.69 (0.04)	4.65 (0.05)	4.58 (0.05)	**0.012**
	Men	4.65 (0.06)	4.61 (0.05)	4.60 (0.08)	0.59	4.73 (0.05)	4.54 (0.08)	4.60 (0.07)	4.61 (0.08)	4.58 (0.08)	0.24
	Women	4.75 (0.07)	4.76 (0.04)	4.65 (0.06)	0.18	4.92 (0.03)	4.65 (0.12)	4.79 (0.05)	4.69 (0.08)	4.59 (0.06)	**0.001**
6. Total dairy and alternatives	Overall	5.31 (0.13)	4.88 (0.10)	4.58 (0.12)	<0.001	5.49 (0.13)	5.17 (0.14)	4.75 (0.17)	4.66 (0.16)	4.37 (0.14)	**<0.001**
	Men	5.82 (0.20)	5.34 (0.14)	4.87 (0.17)	0.001	5.92 (0.18)	5.70 (0.18)	5.23 (0.22)	4.87 (0.26)	4.70 (0.21)	**<0.001**
	Women	4.75 (0.19)	4.35 (0.13)	4.18 (0.16)	0.032	4.99 (0.18)	4.57 (0.22)	4.20 (0.20)	4.41 (0.19)	3.97 (0.18)	**0.001**
7. Fluid intake (total)	Overall	8.54 (0.08)	8.42 (0.07)	8.36 (0.09)	0.15	8.55 (0.11)	8.56 (0.08)	8.45 (0.08)	8.36 (0.09)	8.21 (0.10)	**0.002**
	Men	8.23 (0.13)	8.13 (0.11)	8.02 (0.14)	0.29	8.11 (0.17)	8.20 (0.12)	8.19 (0.13)	7.95 (0.15)	8.20 (0.14)	0.90
	Women	8.92 (0.08)	8.76 (008)	8.68 (0.11)	0.10	9.10 (0.09)	8.97 (0.10)	8.76 (0.11)	8.80 (0.13)	8.30 (0.16)	**<0.001**
7a. Serves per day	Overall	4.01 (0.05)	3.90 (0.04)	3.81 (0.06)	0.023	4.13 (0.06)	3.96 (0.06)	3.83 (0.06)	3.86 (0.07)	3.70 (0.06)	**<0.001**
	Men	3.78 (0.09)	3.66 (0.07)	3.52 (0.10)	0.06	3.84 (0.09)	3.68 (0.08)	3.59 (0.09)	3.53 (0.11)	3.60 (0.10)	**0.015**
	Women	4.29 (0.06)	4.17 (0.05)	4.11 (0.06)	0.06	4.48 (0.05)	4.28 (0.06)	4.11 (0.08)	4.23 (0.08)	3.85 (0.08)	**<0.001**
7b. Mostly water	Overall	4.53 (0.05)	4.52 (0.04)	4.55 (0.05)	0.86	4.42 (0.06)	4.60 (0.04)	4.62 (0.05)	4.50 (0.05)	4.52 (0.07)	0.35
	Men	4.45 (0.08)	4.47 (0.06)	4.50 (0.07)	0.64	4.27 (0.10)	4.52 (0.06)	4.60 (0.07)	4.42 (0.09)	4.60 (0.07)	**0.033**
	Women	4.63 (0.05)	4.59 (0.05)	4.57 (0.06)	0.49	4.62 (0.06)	4.69 (0.06)	4.65 (0.06)	4.57 (0.07)	4.45 (0.11)	0.12
8. Limit discretionary foods	Overall	3.06 (0.18)	2.56 (0.13)	2.49 (0.19)	0.033	2.65 (0.22)	2.52 (0.17)	2.66 (0.17)	2.63 (0.22)	2.99 (0.22)	0.30
	Men	2.79 (0.28)	2.29 (0.18)	1.87 (0.23)	0.022	2.37 (0.33)	2.17 (0.22)	2.24 (0.26)	2.14 (0.30)	2.88 (0.31)	0.46
	Women	3.42 (0.23)	2.86 (0.19)	3.02 (0.26)	0.21	2.97 (0.30)	2.94 (0.25)	3.16 (0.25)	3.15 (0.33)	3.17 (0.31)	0.52
9. Limit saturated fat (total)	Overall	8.74 (0.09)	8.64 (0.07)	8.71 (0.08)	0.73	8.72 (0.08)	8.54 (0.11)	8.66 (0.12)	8.81 (0.10)	8.74 (0.10)	0.34
	Men	8.80 (0.14)	8.50 (0.08)	8.57 (0.14)	0.17	8.62 (0.11)	8.40 (0.17)	8.59 (0.16)	8.81 (0.11)	8.60 (0.17)	0.51
	Women	8.67 (0.12)	8.81 (0.11)	8.88 (0.12)	0.19	8.82 (0.13)	8.70 (0.16)	8.74 (0.16)	8.83 (0.13)	8.88 (0.12)	0.54
9a. Mostly trimmed meat	Overall	4.12 (0.06)	4.04 (0.05)	4.14 (0.07)	0.86	4.07 (0.05)	4.06 (0.07)	4.08 (0.08)	4.21 (0.08)	4.03 (0.09)	0.79
	Men	4.13 (0.10)	3.92 (0.06)	4.02 (0.12)	0.36	3.98 (0.08)	4.00 (0.11)	4.02 (0.12)	4.11 (0.11)	3.87 (0.14)	0.89
	Women	4.11 (0.09)	4.17 (0.07)	4.29 (0.08)	0.10	4.16 (0.09)	4.13 (0.10)	4.15 (0.12)	4.31 (0.09)	4.19 (0.10)	0.52
9b. Mostly low-fat milk	Overall	4.63 (0.04)	4.59 (0.05)	4.54 (0.07)	0.27	4.63 (0.05)	4.51 (0.06)	4.56 (0.06)	4.55 (0.07)	4.73 (0.05)	0.20
	Men	4.66 (0.06)	4.53 (0.06)	4.53 (0.10)	0.20	4.58 (0.07)	4.42 (0.09)	4.56 (0.09)	4.59 (0.09)	4.75 (0.07)	0.09
	Women	4.59 (0.06)	4.67 (0.06)	4.58 (0.08)	0.98	4.68 (0.11)	4.61 (0.07)	4.57 (0.08)	4.51 (0.11)	4.72 (0.07)	0.99
10. Moderate unsaturated-fat	Overall	8.10 (0.16)	8.29 (0.12)	8.54 (0.16)	0.09	8.04 (0.15)	8.35 (0.19)	8.33 (0.14)	8.25 (0.22)	8.58 (0.20)	0.10
	Men	8.83 (0.18)	8.92 (0.13)	9.01 (0.19)	0.53	8.93 (0.16)	8.95 (0.16)	8.86 (0.16)	8.69 (0.25)	9.14 (0.22)	0.94
	Women	7.34 (0.25)	7.62 (0.21)	7.83 (0.28)	0.19	6.99 (0.28)	7.67 (0.35)	7.74 (0.23)	7.69 (0.32)	7.88 (0.34)	0.08
11. Limit added salt (total)	Overall	5.95 (0.11)	6.06 (0.11)	5.61 (0.14)	0.08	6.14 (0.17)	5.95 (0.16)	5.93 (0.15)	5.76 (0.14)	5.77 (0.14)	**0.030**
	Men	5.86 (0.17)	5.89 (0.14)	5.32 (0.28)	0.13	5.93 (0.23)	5.76 (0.20)	5.67 (0.19)	5.78 (0.19)	5.65 (0.21)	0.39
	Women	6.05 (0.17)	6.24 (0.16)	5.95 (0.14)	0.75	6.35 (0.24)	6.17 (0.24)	6.22 (0.20)	5.77 (0.20)	5.94 (0.20)	0.08
11a. During cooking	Overall	2.51 (0.09)	2.78 (0.07)	2.48 (0.09)	0.96	2.84 (0.11)	2.74 (0.11)	2.72 (0.11)	2.44 (0.12)	2.32 (0.11)	**<0.001**
	Men	2.45 (0.13)	2.73 (0.10)	2.45 (0.18)	0.81	2.80 (0.15)	2.62 (0.14)	2.68 (0.15)	2.47 (0.17)	2.28 (0.16)	**0.030**
	Women	2.56 (0.12)	2.84 (0.10)	2.56 (0.12)	0.86	2.86 (0.16)	2.89 (0.16)	2.77 (0.14)	2.43 (0.16)	2.39 (0.16)	**0.004**
11b. Added at the table	Overall	3.44 (0.08)	3.28 (0.06)	3.13 (0.11)	0.031	3.30 (0.10)	3.21 (0.11)	3.21 (0.10)	3.32 (0.10)	3.44 (0.11)	0.23
	Men	3.41 (0.11)	3.17 (0.08)	2.87 (0.17)	0.013	3.13 (0.13)	3.14 (0.13)	2.99 (0.13)	3.31 (0.13)	3.37 (0.15)	0.18
	Women	3.49 (0.10)	3.40 (0.10)	3.39 (0.15)	0.57	3.49 (0.15)	3.29 (0.16)	3.45 (0.14)	3.34 (0.14)	3.54 (0.14)	0.71
12. Limit extra sugar	Overall	6.89 (0.20)	6.45 (0.14)	6.29 (0.21)	0.046	6.49 (0.23)	6.40 (0.23)	6.58 (0.19)	6.57 (0.23)	6.67 (0.23)	0.53
	Men	6.68 (0.33)	6.07 (0.16)	6.25 (0.32)	0.33	6.02 (0.33)	6.25 (0.37)	6.26 (0.26)	6.13 (0.34)	6.84 (0.34)	0.26
	Women	7.14 (0.28)	6.92 (0.22)	6.42 (0.29)	0.10	7.09 (0.35)	6.57 90.31)	6.93 (0.31)	7.07 (0.33)	6.61 (0.26)	0.59
13. Limit alcohol	Overall	8.67 (0.14)	8.45 (0.10)	8.69 (0.16)	0.97	8.20 (0.14)	8.42 (0.17)	8.58 (0.15)	8.85 (0.17)	8.91 (0.16)	**0.003**
	Men	8.05 (0.23)	8.02 (0.13)	8.21 (0.29)	0.70	7.57 (0.23)	7.90 (0.27)	8.10 (0.25)	8.49 (0.25)	8.64 (0.28)	**0.006**
	Women	9.35 (0.14)	8.90 (0.17)	9.25 (0.15)	0.54	8.97 (0.19)	9.02 (0.21)	9.13 (0.22)	9.29 (0.17)	9.27 (0.18)	0.20

DGI scores could range between 0 and 130; Q, quintile; Values represent predictive margins and SE. ^1^ Denotes group of lowest socioeconomic disadvantage; ^2^ Linear regression analyses were adjusted for age, sex (not when used to stratify), urban/rural location, smoking and ratio of energy intake to predicted energy expenditure (EI:EE).

**Table 3:** Total energy and nutrient intakes by area-level disadvantage in men (*n* = 2356) and women (*n* = 2519) from the 2011-13 Australian National Nutrition and Physical Activity Survey.

Energy/nutrient	Sample	*n*	Area-Level Disadvantage					
			Least Disadvantaged 20% ^1^	Q2	Q3	Q4	Most Disadvantaged 20%	*p*-*Trend^2^*
Energy intake (kJ/day)	Overall	4875	8655 (47.9)	8697 (60.8)	8582 (46.9)	8639 (45.9)	8654 (55)	0.75
	Men	2356	9725 (76.5)	9887 (90.8)	9733 (58.1)	9705 (62.1)	9667 (74.9)	0.21
	Women	2519	7456 (50.3)	7259 (45.6)	7316 (55)	7452 (64.6)	7450 (64.2)	0.47
Protein intake (%E/day)	Overall	4875	18.9 (0.21)	18.9 (0.25)	18.5 (0.22)	18.5 (0.28)	18.4 (0.27)	0.06
	Men	2356	18.9 (0.26)	18.7 (0.34)	18.5 (0.31)	18.6 (0.42)	18.4 (0.34)	0.24
	Women	2519	19 (0.32)	19.1 (0.36)	18.5 (0.35)	18.3 (0.33)	18.4 (0.38)	0.09
Total fat (%E/day)	Overall	4875	31.2 (0.35)	31.5 (0.36)	30.4 (0.27)	30.6 (0.29)	30.5 (0.44)	**0.049**
	Men	2356	30.6 (0.51)	31.4 (0.44)	30.4 (0.53)	30.2 (0.42)	30.5 (0.63)	0.48
	Women	2519	32 (0.47)	31.6 (0.52)	30.5 (0.39)	30.9 (0.39)	30.4 (0.55)	**0.016**
Saturated fat intake (%E/day)	Overall	4875	11.7 (0.18)	11.6 (0.19)	11.4 (0.17)	11.4 (0.15)	11.3 (0.2)	0.05
	Men	2356	11.7 (0.25)	11.8 (0.24)	11.4 (0.22)	11.2 (0.2)	11.3 (0.3)	0.09
	Women	2519	11.8 (0.25)	11.3 (0.27)	11.5 (0.25)	11.6 (0.23)	11.2 (0.25)	0.33
Trans fat intake (%E/day)	Overall	4875	0.58 (0.012)	0.56 (0.016)	0.58 (0.016)	0.56 (0.011)	0.56 (0.015)	0.34
	Men	2356	0.59 (0.019)	0.57 (0.019)	0.59 (0.019)	0.56 (0.013)	0.58 (0.023)	0.51
	Women	2519	0.57 (0.015)	0.54 (0.025)	0.58 (0.024)	0.56 (0.018)	0.54 (0.019)	0.48
Mono-unsaturated fat intake (%E/day)	Overall	4875	11.9 (0.17)	12.2 (0.17)	11.6 (0.13)	11.7 (0.13)	11.7 (0.19)	0.10
	Men	2356	11.6 (0.24)	12.1 (0.22)	11.7 (0.24)	11.7 (0.19)	11.8 (0.29)	0.79
	Women	2519	12.4 (0.21)	12.2 (0.25)	11.5 (0.2)	11.7 (0.17)	11.6 (0.25)	**0.005**
Poly-unsaturated fat intake (%E/day)	Overall	4875	4.8 (0.08)	5 (0.13)	4.7 (0.08)	4.8 (0.08)	4.8 (0.1)	0.60
	Men	2356	4.6 (0.11)	4.8 (0.19)	4.7 (0.15)	4.7 (0.11)	4.7 (0.14)	0.70
	Women	2519	5.1 (0.13)	5.2 (0.19)	4.7 (0.11)	4.9 (0.13)	4.9 (0.16)	0.27
Carbohydrate intake (%E/day)	Overall	4875	41.4 (0.44)	42.3 (0.47)	44.2 (0.42)	44.2 (0.39)	44.3 (0.5)	**<0.001**
	Men	2356	41.8 (0.53)	42.2 (0.56)	44 (0.77)	43.6 (0.55)	43.5 (0.66)	**0.011**
	Women	2519	40.9 (0.63)	42.3 (0.68)	44.4 (0.64)	44.8 (0.53)	45.2 (0.67)	**<0.001**
Total sugars intake (%E/day)	Overall	4875	17.7 (0.38)	18.6 (0.35)	19.7 (0.36)	19.3 (0.35)	18.6 (0.41)	**0.027**
	Men	2356	17.4 (0.45)	18.1 (0.5)	19 (0.49)	18.9 (0.49)	17.8 (0.68)	0.29
	Women	2519	18.1 (0.47)	19.1 (0.44)	20.6 (0.55)	19.7 (0.39)	19.4 (0.51)	**0.02**
Fibre intake (g/MJ)	Overall	4875	2.7 (0.04)	2.8 (0.05)	2.8 (0.05)	2.8 (0.04)	2.8 (0.04)	0.58
	Men	2356	2.6 (0.07)	2.7 (0.06)	2.6 (0.08)	2.6 (0.05)	2.6 (0.06)	0.81
	Women	2519	2.9 (0.07)	3 (0.07)	3 (0.06)	3 (0.06)	2.9 (0.07)	0.64
Sodium intake (g/MJ)	Overall	4875	284.4 (3.95)	289.7 (5.7)	280.8 (4.27)	275.2 (4.06)	289.7 (4.94)	0.76
	Men	2356	288.1 (6.06)	286.3 (7.31)	284.1 (6.6)	272.1 (5.67)	289.4 (6.86)	0.51
	Women	2519	280.2 (5.44)	294.2 (8.21)	277.3 (6.34)	278.3 (6.03)	289.9 (6.12)	0.77

Area-level disadvantage was assessed using SEIFA (Socio-Economic Index for Areas); Q, quintile; Values represent predictive margins and SE. ^1^ Denotes group of lowest socioeconomic disadvantage; ^2^ Linear regression analyses were adjusted for age, sex (for total only), urban/rural location, smoking, and ratio of energy intake to predicted energy expenditure (EI:EE).

**Table 4:** Total energy and nutrient intakes by education and income in men (*n* = 2356) and women (*n* = 2519) from the 2011-13 Australian National Nutrition and Physical Activity Survey.

Energy/nutrient	Sample	*n*	Education				Gross Equivalised Income of Household (Weekly)					
			University Qualification ^1^	High-School/Diploma	Some High-School or Less	*p*-*Trend* ^2^	Highest 20% (≥$1152) ^1^	Q2 ($959–1151)	Q3 ($639–958)	Q4 ($399–638)	Lowest 20% (Below Poverty Line; ≤$398)	*p*-*Trend* ^2^
Energy intake (kJ/day)	Overall	4875	8646 (44.5)	8626 (28.5)	8677 (53.3)	0.72	8795 (38.4)	8728 (50.5)	8626 (53.9)	8541 (50.9)	8465 (45.2)	**<0.001**
	Men	2356	9833 (68.3)	9716 (41.1)	9707 (82.5)	0.20	9959 (61.4)	9808 (59.9)	9748 (83.2)	9556 (81.6)	9490 (70.1)	**<0.001**
	Women	2519	7346 (44.1)	7422 (34.4)	7382 (60.1)	0.56	7456 (49)	7511 (74.4)	7374 (44.7)	7340 (55.2)	7251 (54.1)	**0.002**
Protein intake (%E/day)	Overall	4875	18.3 (0.21)	18.8 (0.14)	18.8 (0.23)	0.13	19.1 (0.24)	18.6 (0.25)	18.7 (0.24)	18.7 (0.26)	18.1 (0.25)	0.038
	Men	2356	18.3 (0.32)	18.7 (0.17)	18.8 (0.29)	0.29	19 (0.31)	18.6 (0.3)	18.7 (0.31)	18.4 (0.42)	18.4 (0.33)	0.22
	Women	2519	18.4 (0.25)	18.8 (0.26)	18.7 (0.31)	0.38	19.2 (0.33)	18.6 (0.34)	18.7 (0.4)	19 (0.29)	17.9 (0.33)	0.06
Total fat (%E/day)	Overall	4875	31.3 (0.34)	30.6 (0.2)	30.7 (0.36)	0.14	31.3 (0.34)	30.2 (0.31)	30.7 (0.28)	31 (0.35)	31 (0.35)	0.78
	Men	2356	31 (0.53)	30.3 (0.26)	30.8 (0.5)	0.67	31.1 (0.43)	29.7 (0.42)	30.3 (0.31)	31 (0.55)	31.1 (0.6)	0.63
	Women	2519	31.6 (0.41)	31 (0.33)	30.6 (0.42)	0.07	31.7 (0.54)	30.8 (0.46)	31.2 (0.4)	31 (0.5)	30.8 (0.48)	0.37
Saturated fat intake (%E/day)	Overall	4875	11.6 (0.16)	11.4 (0.1)	11.7 (0.18)	0.87	11.4 (0.15)	11.3 (0.14)	11.5 (0.15)	11.7 (0.19)	11.6 (0.19)	0.15
	Men	2356	11.6 (0.24)	11.4 (0.14)	11.6 (0.19)	0.77	11.5 (0.18)	11.2 (0.18)	11.4 (0.17)	11.8 (0.24)	11.7 (0.28)	0.22
	Women	2519	11.5 (0.21)	11.3 (0.17)	11.7 (0.25)	0.60	11.3 (0.23)	11.4 (0.26)	11.6 (0.24)	11.6 (0.25)	11.6 (0.25)	0.44
Trans fat intake (%E/day)	Overall	4875	0.56 (0.009)	0.56 (0.008)	0.61 (0.015)	0.002	0.56 (0.011)	0.56 (0.012)	0.56 (0.011)	0.58 (0.015)	0.6 (0.016)	**0.037**
	Men	2356	0.56 (0.013)	0.57 (0.011)	0.62 (0.019)	0.013	0.58 (0.015)	0.57 (0.019)	0.56 (0.013)	0.58 (0.021)	0.6 (0.023)	0.42
	Women	2519	0.55 (0.012)	0.54 (0.013)	0.6 (0.021)	0.019	0.53 (0.014)	0.54 (0.016)	0.56 (0.018)	0.57 (0.021)	0.59 (0.023)	**0.013**
Mono-unsaturated fat intake (%E/day)	Overall	4875	12 (0.16)	11.8 (0.11)	11.7 (0.17)	0.17	12.1 (0.19)	11.5 (0.15)	11.8 (0.12)	11.8 (0.15)	11.8 (0.15)	0.39
	Men	2356	11.9 (0.23)	11.7 (0.15)	11.8 (0.25)	0.79	12 (0.23)	11.4 (0.21)	11.7 (0.17)	11.8 (0.24)	12.1 (0.28)	0.66
	Women	2519	12.1 (0.2)	12 (0.15)	11.5 (0.19)	0.049	12.3 (0.28)	11.8 (0.19)	11.9 (0.17)	11.8 (0.24)	11.6 (0.19)	**0.047**
Poly-unsaturated fat intake (%E/day)	Overall	4875	5 (0.08)	4.8 (0.07)	4.7 (0.1)	0.020	5 (0.1)	4.7 (0.08)	4.8 (0.09)	4.8 (0.09)	4.8 (0.11)	0.11
	Men	2356	4.8 (0.11)	4.6 (0.09)	4.7 (0.14)	0.57	4.9 (0.17)	4.5 (0.12)	4.7 (0.12)	4.7 (0.14)	4.6 (0.15)	0.26
	Women	2519	5.2 (0.1)	5.1 (0.1)	4.6 (0.13)	0.005	5.2 (0.14)	4.9 (0.11)	4.9 (0.14)	4.9 (0.14)	4.9 (0.18)	0.39
Carbohydrate intake (%E/day)	Overall	4875	42.8 (0.42)	43 (0.24)	44.1 (0.43)	0.03	40.8 (0.48)	43.3 (0.46)	43.6 (0.43)	44 (0.39)	45.1 (0.44)	**<0.001**
	Men	2356	42.6 (0.58)	42.9 (0.36)	43.7 (0.58)	0.19	40.6 (0.51)	43.4 (0.61)	43.6 (0.59)	44 (0.61)	44.6 (0.67)	**<0.001**
	Women	2519	43 (0.49)	43.1 (0.35)	44.6 (0.58)	0.035	41.2 (0.8)	43.2 (0.6)	43.6 (0.67)	44 (0.55)	45.5 (0.57)	**<0.001**
Total sugars intake (%E/day)	Overall	4875	18.2 (0.26)	18.8 (0.22)	19.4 (0.32)	0.004	18.1 (0.34)	18.9 (0.31)	19 (0.4)	19.2 (0.4)	18.8 (0.38)	0.13
	Men	2356	17.2 (0.4)	18.6 (0.27)	18.7 (0.54)	0.017	17.1 (0.4)	18.6 (0.53)	18.8 (0.51)	19.1 (0.62)	17.8 (0.58)	0.17
	Women	2519	19.3 (0.33)	18.9 (0.3)	20.2 (0.48)	0.19	19.4 (0.5)	19.2 (0.38)	19.3 (0.5)	19.4 (0.46)	19.7 (0.54)	0.55
Fibre intake (g/MJ)	Overall	4875	2.9 (0.04)	2.7 (0.03)	2.7 (0.04)	0.005	2.8 (0.04)	2.8 (0.05)	2.7 (0.03)	2.8 (0.04)	2.8 (0.05)	1.00
	Men	2356	2.7 (0.06)	2.6 (0.04)	2.6 (0.05)	0.031	2.6 (0.06)	2.7 (0.06)	2.6 (0.05)	2.7 (0.06)	2.6 (0.07)	0.53
	Women	2519	3.1 (0.06)	2.9 (0.04)	2.9 (0.06)	0.032	3 (0.06)	3 (0.07)	2.9 (0.05)	2.9 (0.06)	3 (0.07)	0.45
Sodium intake (g/MJ)	Overall	4875	282.5 (3.7)	285.5 (2.47)	281.6 (4.22)	0.93	282.6 (4.16)	282.5 (4.45)	287.4 (5.41)	282.4 (4.17)	283.7 (5.38)	0.83
	Men	2356	286.4 (4.89)	282.7 (3.56)	284 (6.34)	0.73	286.1 (5.65)	279 (6.57)	289.5 (7.52)	286.3 (5.5)	277.2 (7.73)	0.72
	Women	2519	278.8 (5.04)	289.4 (3.99)	279.9 (6.19)	0.78	277.4 (5.9)	286.8 (6)	285.2 (6.5)	278.1 (7.1)	289.1 (6.1)	0.34

Q, quintile; Values represent predictive margins and SE. ^1^ Denotes group of lowest socioeconomic disadvantage; ^2^ Linear regression analyses were adjusted for age, sex (for total only), urban/rural location, smoking and ratio of energy intake to predicted energy expenditure (EI:EE)

## Supplementary Materials

The following are available online at http://www.mdpi.com/2072-6643/9/10/1092/s1. Figure S1: Flow diagram of subjects included in the cross-sectional analysis of the Australian National Nutrition and Physical Activity Survey; Table S1: Components and scoring methods of the Dietary Guideline Index (DGI); Tables S2 and S3: Participant characteristics in adults from the National Nutrition and Physical Activity Survey; Table S4: Participant characteristics of valid-energy reporters and the total sample in adults from the Australian National Nutrition and Physical Activity Survey.
