# Supplementary material for: Socioeconomic Inequities in Diet Quality and Nutrient Intakes among Australian Adults: Findings from a Nationally Representative Cross-Sectional Study
Source: Nutrients. 2017 Oct 4;9(10):1092. doi: 10.3390/nu9101092 (PMC5691709; doi:10.3390/nu9101092)
Supplement: Supplementary file 1 [file nutrients-09-01092-s001.pdf]

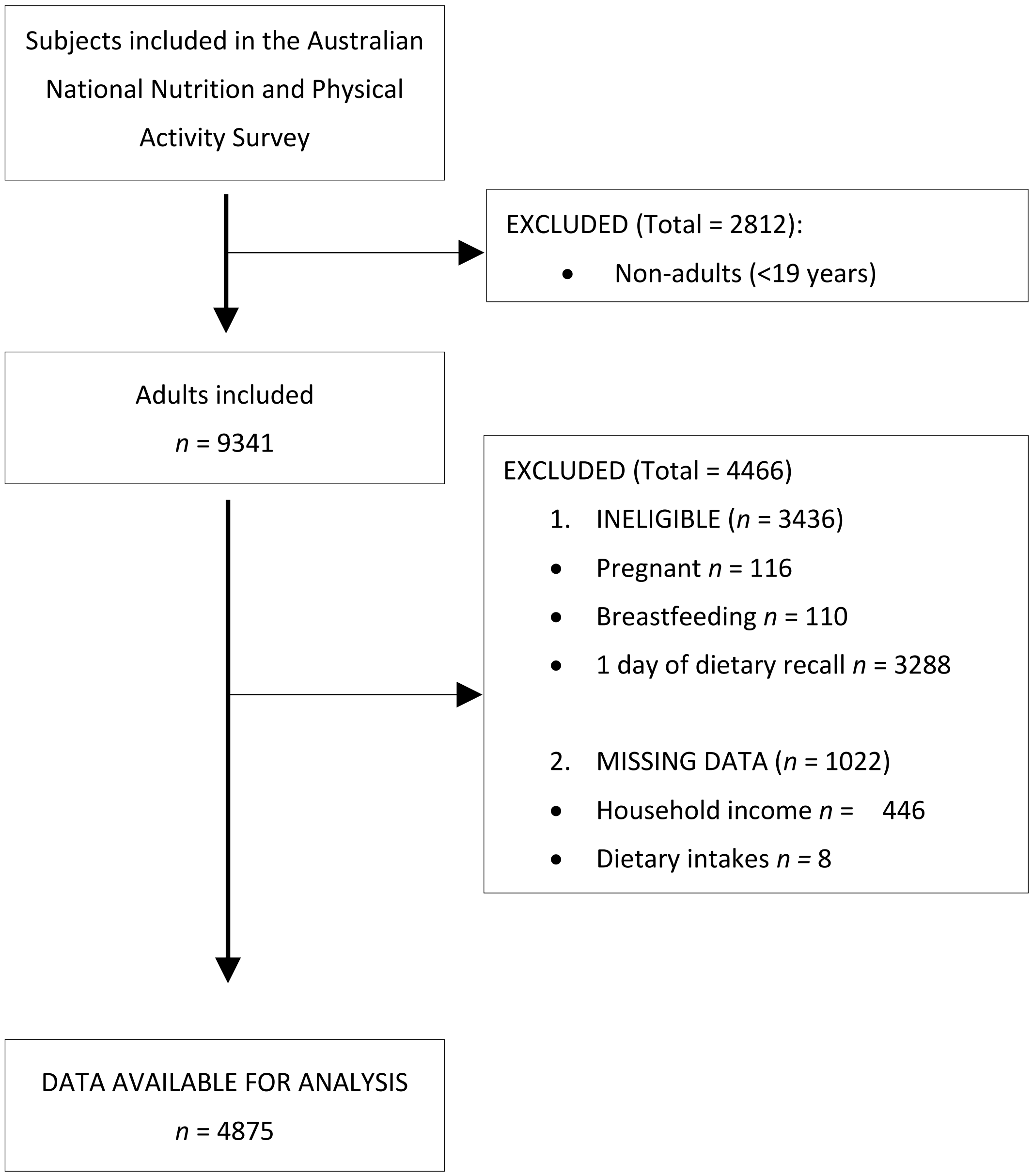

Supplemental Figure 1. Flow diagram of subjects included in the cross-sectional analysis of the Australian National Nutrition and Physical Activity Survey

Supplemental Table 1 Components and scoring methods of the Dietary Guideline Index (DGI)

Dietary Guideline	Indicator and description	Criteria for maximum score[1]	Criteria for minimum score (0)	Maximum Score
Guidelines for adequate intake				
1. Enjoy a wide variety of nutritious foods	Food variety [2]: proportion of food from each of the 5 core food groups eaten at least one serve per week	100%	0%	10
2. Plenty of vegetables	Total vegetable intake: servings of vegetables per day	19-50 y: M ≥ 6, F ≥ 5<br>51-70 y: M ≥ 5.5, F ≥ 5<br>>70 y: M ≥ 5, F ≥ 5	0	10
3. Fruit	Total fruit intake: servings of fruit per day	≥ 2	0	10
4. Grain (cereal) foods	Total cereal intake: servings of grains per day	19-50 y: M ≥ 6, F ≥ 6<br>51-70 y: M ≥ 6, F ≥ 4<br>>70 y: M ≥ 4.5, F ≥ 3	0	5
	Wholegrain or high fibre cereals: proportion of wholegrain bread to white bread intake per day	≥ 50% wholemeal bread	0%	5
5. Lean meat and poultry, fish, eggs, nuts and seeds, and legumes/beans	Total lean meat and alternative: servings per day	19-50 y: M ≥ 3, F ≥ 2.5<br>51-70 y: M ≥ 2.5, F ≥ 2<br>>70 y: M ≥ 2.5, F ≥ 2	0	5
	Lean meat: proportion of lean meats and alternatives to total meat and alternatives per day	100%	0%	5
6. Milk, yoghurt, cheese and/or their alternatives[3]	Total dairy and alternative: servings per day	19-50 y: M ≥ 2.5, F ≥ 2.5<br>51-70 y: M ≥ 2.5, F ≥ 4	0	10

		>70 y: M ≥ 3.5, F ≥ 4		
7. Drink plenty of water	Total beverage intake[4]: servings per day	M ≥ 10; F ≥ 8	0	5
	Water[5]: proportion of water to total beverage intake per day	≥ 50%	0%	5
Guidelines to limit or moderate intake				
8. Limit intake of foods containing saturated fat, added salt, added sugars and alcohol	Limit discretionary foods	M ≤ 3; F ≤ 2.5	M > 3; F > 2.5	10
9. Limit intake of foods high in saturated fat	Trim meat: proportion of trimmed meat to total meat	≥ 50%	0%	5
	Choose reduced-fat milk: proportion of reduced-fat milk to total milk intake per day	≥ 50% reduced-fat milk	0%	5
10. Small allowance of unsaturated oils, fats or spreads	Unsaturated spreads and oils: servings per day	19-50 y: M ≤ 4, F ≤ 2<br>51-70 y: M ≤ 4, F ≤ 2<br>>70 y: M ≤ 2, F ≤ 2	M > 4; F > 2	10
11. Limit intake of foods and drinks containing added salt	Salt use: salt added during cooking	Never or rarely	Usually	5
	Salt use: salt added during the meal	Never or rarely	Usually	5
12. Limit intake of foods and drinks containing added sugars	Limit extra sugar[6]: servings per day	M ≤ 1.5; F ≤ 1.25	M > 1.5; F > 1.25	10
13. If you choose to drink alcohol, limit intake	Limit alcohol: servings per day	≤ 2	> 2	10

1. Criteria for maximum scores were derived from the Australian Dietary Guidelines unless otherwise noted.

2. Food variety was measured and scored using a similar method to the Recommended Food Score.

3. Choosing reduced fat dairy is captured in the “Limit intake of foods high in saturated fat” component.

4. The Eat for Health Australian Dietary Guidelines do not have specific recommendations for beverage consumption and recommended the guidelines found in the Nutrient Reference Values for Australia and New Zealand (30).

5. The proportion of water to total beverage intake was derived from US beverage guidelines.

6. Since added sugar intake is not recommended there are no cut-off values for number or recommended serves, instead half of the maximum discretionary food cut-off were used consistent with the original DGI.

Supplemental Table 2. Participant characteristics according to categories of the area-level disadvantage in adults from the National Nutrition and Physical Activity Survey.

	Total	Area-level disadvantage					
		Least disadvantaged 20%[1]	Q2	Q3	Q4	Most disadvantaged 20%	P-*trend*
n	4875	1152	887	937	994	905	
Age, y	47.1 (0.19)	46.3 (0.68)	45.7 (0.75)	46.6 (0.84)	47.0 (0.85)	50.4 (0.97)	0.001
Women, %	46.7	47.0	44.4	48.2	45.5	48.2	0.75
Location, %							
Urban	70.0	83.6	85.8	64.7	60.3	54.2	<0.001
Rural	20.1	7.4	14.2	25.2	24.1	30.9	
Other	9.9	9.0	0	10.1	15.7	14.9	
Country of birth, %							
Australia	68.9	66.0	65.4	72.6	71.6	69.0	0.047
English speaking country	12.1	14.6	14.0	12.3	10.9	8.0	
Other	19.0	19.4	20.6	15.1	17.5	23.0	
Current smoker, %							
Current smoker	16.4	7.9	13.6	18.7	19.6	23.2	<0.001
Ex-smoker	32.9	34.4	33.4	33.1	33.8	29.3	
Never smoked	50.7	57.7	53.0	48.3	46.6	47.5	

DGI score	80.3 (0.33)	81.9 (0.71)	81.9 (0.75)	80.1 (0.84)	79.6 (0.72)	77.7 (0.70)	<0.001
Nutrient intakes							
Total energy, kJ/d	8644 (51.3)	8754 (122.9)	8866 (180.5)	8629 (108.6)	8593 (144.9)	8356 (124.9)	0.039
Total fat, %E	30.8 (0.16)	31.3 (0.34)	31.4 (0.35)	30.5 (0.26)	30.6 (0.29)	30.4 (0.42)	0.028
Saturated fat, %E	11.5 (0.08)	11.7 (0.18)	11.5 (0.20)	11.5 (0.17)	11.5 (0.15)	11.4 (0.20)	0.30
Mono-unsaturated fat, %E	11.8 (0.08)	12.0 (0.16)	12.2 (0.17)	11.7 (0.13)	11.7 (0.13)	11.6 (0.18)	0.017
Poly-unsaturated fat, %E	4.8 (0.05)	4.9 (0.07)	5.1 (0.13)	4.7 (0.08)	4.8 (0.08)	4.7 (0.10)	0.11
Trans fat, %E	0.57 (0.01)	0.57 (0.01)	0.55 (0.02)	0.59 (0.02)	0.56 (0.01)	0.58 (0.01)	0.55
Carbohydrates, %E	43.2 (0.19)	41.7 (0.46)	42.6 (0.48)	44.1 (0.44)	43.9 (0.42)	43.9 (0.50)	<0.001
Total sugars, %E	18.8 (0.02)	17.5 (0.37)	18.4 (0.34)	19.8 (0.35)	19.4 (0.36)	18.8 (0.40)	0.002
Protein, %E	18.6 (0.11)	18.9 (0.20)	18.8 (0.25)	18.4 (0.24)	18.6 (0.30)	18.5 (0.26)	0.13
Fibre density, g/MJ	2.78 (0.02)	2.74 (0.04)	2.84 (0.05)	2.78 (0.06)	2.76 (0.04)	2.78 (0.05)	0.92
Sodium density, mg/MJ	283.7 (1.81)	282.0 (3.94)	287.6 (5.73)	281.3 (4.53)	277.4 (4.46)	291.5 (5.03)	0.55

Area-level disadvantage was assessed using SEIFA (Socio-Economic Index for Areas); Q, quintile. Values represent means and SE or percentages. 1, Denotes group of lowest socioeconomic disadvantage

	Education level				Gross equivalised income of household (weekly)					
	University qualification[1]	High-school/ certificate/ diploma	Some high-school or less	P-*trend*	Highest 20% (>$1152)[1]	Q2 ($959-1151)	Q3 ($639-958)	Q4 ($399-638)	Lowest 20% (below poverty line; <$398)	P-*trend*
n	1346	2260	1269		1101	1049	914	861	950	
Age, y	42.2	44.8	57.4	<0.001	43.4	42.5	44.2	52.2	56.3	<0.001
Women, %	50.3	40.4	55.4	<0.001	40.7	45.1	46.7	48.3	54.8	0.001
Location, %										
Urban	83.0	67.9	59.2	<0.001	80.6	74.1	64.9	64.5	62.7	<0.001
Rural	12.2	21.7	26.0		13.8	15.3	24.4	24.3	24.9	
Other	4.7	10.5	14.9		5.6	10.6	10.7	11.3	12.5	
Country of birth, %										
Australia	60.0	69.9	77.2	<0.001	71.5	68.4	69.2	68.8	66.2	0.003
English speaking country	11.6	13.9	8.9		15.5	12.2	12.1	10.6	9.0	
Other	28.4	16.1	13.9		13.0	19.4	18.8	20.6	24.8	
Current smoker, %										
Current smoker	8.1	17.8	23.1	<0.001	12.2	14.5	15.3	20.7	21.1	0.007
Ex-smoker	27.6	34.6	35.5		31.1	35.8	31.8	33.2	32.5	
Never smoked	64.3	47.6	41.4		56.6	49.7	52.9	46.1	46.4	
DGI score	83.0 (0.61)	79.3 (0.45)	79.2 (0.50)	<0.001	81.6 (0.73)	80.2 (0.76)	79.5 (0.60)	80.1 (0.62)	79.9 (0.69)	0.102
Nutrient intakes										
Total energy, kJ/d	9030 (119.6)	8750 (71.4)	7985 (128.2)	<0.001	9148 (138.9)	9012 (136.8)	8682 (157.2)	8425 (135.5)	7706 (139.8)	<0.001
Total fat, %E	31.5 (0.33)	30.6 (0.20)	30.5 (0.34)	0.017	31.4 (0.34)	30.4 (0.31)	30.8 (0.30)	30.9 (0.37)	30.6 (0.34)	0.28

Saturated fat, %E	11.6 (0.16)	11.4 (0.11)	11.6 (0.18)	0.97	11.4 (0.16)	11.4 (0.15)	11.5 (0.16)	11.7 (0.19)	11.5 (0.19)	0.29
Mono-unsaturated fat, %E	12.1 (0.15)	11.8 (0.11)	11.5 (0.16)	0.010	12.2 (0.18)	11.6 (0.15)	11.8 (0.13)	11.7 (0.16)	11.6 (0.14)	0.041
Poly-unsaturated fat, %E	5.0 (0.08)	4.8 (0.07)	4.6 (0.09)	<0.001	5.1 (0.11)	4.7 (0.08)	4.8 (0.10)	4.8 (0.09)	4.7 (0.10)	0.026
Trans fat, %E	0.55 (0.01)	0.56 (0.01)	0.61 (0.01)	<0.001	0.55 (0.01)	0.56 (0.01)	0.56 (0.01)	0.59 (0.01)	0.60 (0.02)	0.009
Carbohydrates, %E	43.4 (0.41)	43.0 (0.24)	43.4 (0.40)	0.99	41.3 (0.44)	43.5 (0.49)	43.8 (0.44)	43.5 (0.40)	44.5 (0.44)	<0.001
Total sugars, %E	18.2 (0.25)	18.7 (0.22)	19.5 (0.29)	0.001	18.0 (0.33)	18.8 (0.31)	19.1 (0.39)	19.3 (0.39)	18.9 (0.35)	0.023
Protein, %E	18.2 (0.20)	18.8 (0.15)	18.8 (0.21)	0.028	19.0 (0.25)	18.5 (0.26)	18.7 (0.27)	18.6 (0.26)	18.3 (0.23)	0.14
Fibre density, g/MJ	2.86 (0.04)	2.69 (0.03)	2.86 (0.04)	0.87	2.72 (0.04)	2.77 (0.05)	2.68 (0.03)	2.85 (0.05)	2.92 (0.05)	0.002
Sodium density, mg/MJ	280.3 (3.52)	287.7 (2.54)	2.79.7 (4.06)	0.98	282.0 (4.21)	283.6 (4.57)	289.7 (5.40)	280.1 (4.07)	282.5 (5.01)	0.92

Supplemental Table 3. Participant characteristics according to educational level and income in adults from the Australian National Nutrition and Physical Activity Survey

Values represent means and SE or percentages. 1, Denotes group of lowest socioeconomic disadvantage

Supplemental Table 4. Participant characteristics of valid-energy reporters and the total sample in adults from the Australian National Nutrition and Physical Activity Survey

	Total sample	Plausible energy reporters
n	4875	3320
Age, y	47.1 (0.19)	47.2 (0.33)
Women, %	46.7	46.7
Location, %		
Urban	70.0	69.6
Rural	20.1	21.1
Other	9.9	9.3
Country of birth		
Australia	68.9	70.3
English speaking country	12.1	12.4
Other	19.0	17.3
Current smoker, %		
Current smoker	16.4	14.3
Ex-smoker	32.9	33.4
Never smoked	50.7	52.3
DGI-2013 score	80.3 (0.33)	80.1 (0.40)
Macronutrient intakes		
Total Energy, kJ/d	8644 (51.3)	9130 (43.2)
Total fat, %E	30.8 (0.16)	31.2
Saturated fat, %E	11.5 (0.08)	11.7
Mono-unsaturated fat, %E	11.8 (0.08)	11.9
Poly-unsaturated fat, %E	4.8 (0.05)	4.8
Trans fat, %E	0.57 (0.01)	0.58
Carbohydrates, %E	43.2 (0.19)	43.0
Total sugars, %E	18.8 (0.02)	18.8
Protein, %E	18.6 (0.11)	18.2
Fibre density, g/MJ	2.78 (0.02)	2.73 (0.024)
Sodium density, mg/MJ	283.7 (1.81)	277.3 (2.22)
